# Biology of Interleukin-17 and Its Pathophysiological Significance in Sepsis

**DOI:** 10.3389/fimmu.2020.01558

**Published:** 2020-07-28

**Authors:** Yun Ge, Man Huang, Yong-ming Yao

**Affiliations:** ^1^Department of General Intensive Care Unit, The Second Affiliated Hospital of Zhejiang University School of Medicine, Hangzhou, China; ^2^Trauma Research Center, Fourth Medical Center and Medical Innovation Research Department of the Chinese PLA General Hospital, Beijing, China

**Keywords:** interleukin-17, cytokine, sepsis, inflammatory response, immune response

## Abstract

The interleukin (IL)-17 family includes six structure-related cytokines (A–F). To date, majority of studies have focused on IL-17A. IL-17A plays a pivotal role in various infectious diseases, inflammatory and autoimmune disorders, and cancer. Several recent studies have indicated that IL-17A is a biomarker as well as a therapeutic target in sepsis. In the current review, we summarize the biological functions of IL-17, including IL-17-mediated responses and signal transduction pathways, with particular emphasis on clinical relevance to sepsis.

## Introduction

**Figure 1:**
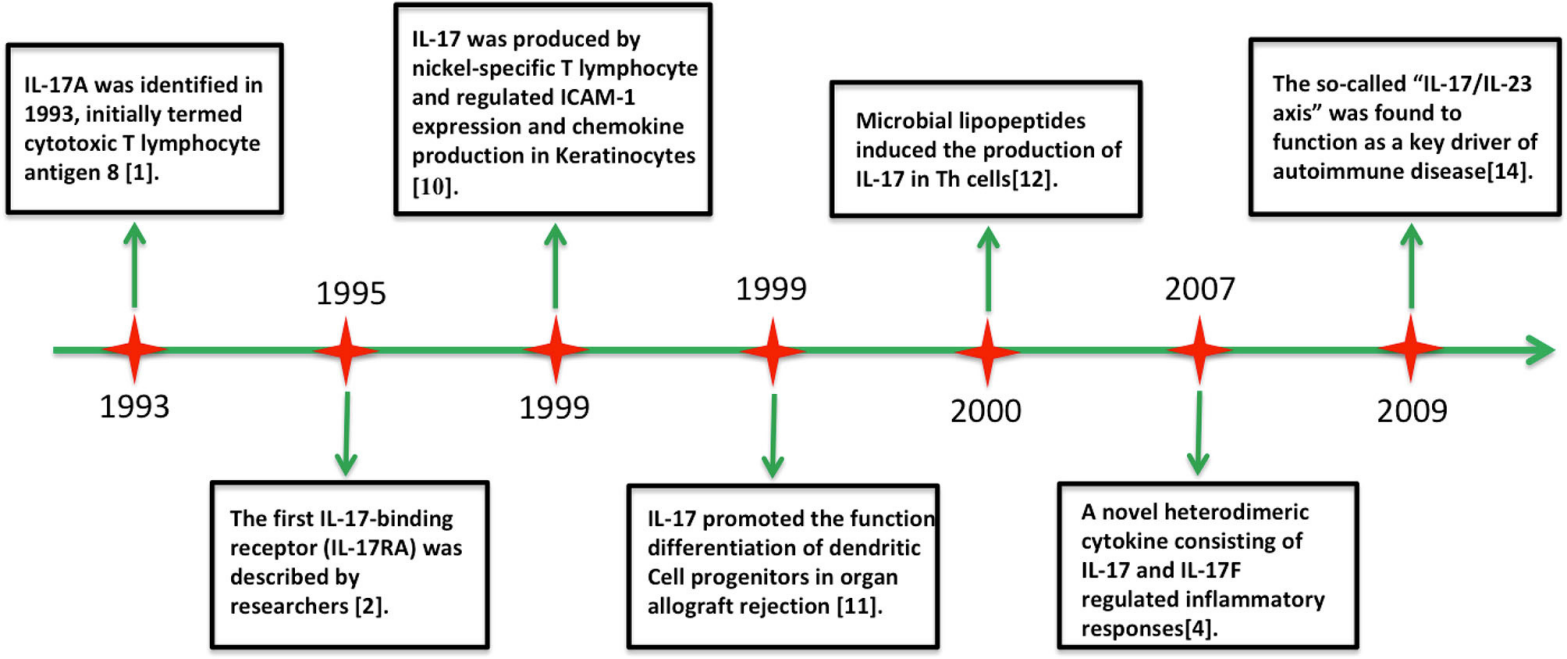
Timeline of advances in the research on interleukin-17 cytokine.

The interleukin (IL)-17 family includes six structure-related cytokines (A–F; Figure 1). IL-17A, the first discovered member of the IL-17 family, was cloned in 1993 and originally termed as cytotoxic T lymphocyte antigen 8 (1). Unexpectedly, IL-17A shares sequence homology with an open reading frame in *Herpesvirus saimiri* (1). IL-17-binding receptor (IL-17RA) was subsequently identified in 1995 (2). Screens for homologous genes caused the discovery of other five highly conserved homologous members of the IL-17 family (IL-17B to IL-17F) (3, 4). IL-17F shows high homology with IL-17A, whereas IL-17E (also known as IL-25) shows only 16% sequence homology with IL-17A (5, 6). Analogous to platelet-derived growth factor and nerve growth factor, these molecules adopt a cysteine knot fold (3, 4). IL-17B and IL-17D have been shown to induce the secretion of chemokines and proinflammatory cytokines, but their biological actions remain scarcely explored (7–9). In contrast, the proinflammatory properties of IL-17A and IL-17F are well characterized (5, 6). Additionally, the IL-17 receptor family includes five cytokine receptors (IL-17RA to IL-17RE) characterized by a shared cytoplasmic motif named the SEF/IL-17R (SEFIR) (5).

IL-17A expression was first reported in T helper (Th) cells (10, 11). IL-17A is recognized as a hallmark molecule of CD4^+^ T helper 17 (Th17) cells, which characteristically express the transcription factor RAR-related orphan receptor γ (RORγt) (12). The development of Th17 cells, including clonal expansion and phenotype stabilization of IL-17A production, is dependent on IL-23 (13). Mechanistically, IL-23 expands the Th17 cell population by upregulating signal transducers and activators of transcription (STAT)-triggered RORγt and subsequent promotion of IL-17A release (14). Thus, IL-23 is recognized as a potent inducer of IL-17A. Recent studies suggest that the so-called “IL-17/IL-23 axis” is a key element in inflammation and is involved in the immune responses to fungal and bacterial infection and autoimmune diseases (12, 13).

**Figure 2:**
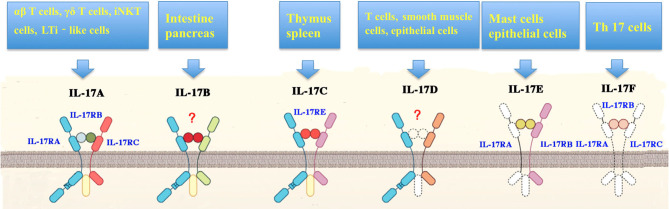
Cellular sources and receptors of interleukin-17. Interleukin (IL)-17 family includes isoforms A to F. IL-17 can be induced by a variety of cells including T helper (Th) 17 cells, γδT cells, natural killer T (NKT) cells, group 3 innate lymphoid cells (ILC3s), CD8^+^ (Tc17) cells, neutrophils, microglia, mast cells, myeloid cells in the lung and kidneys, and Paneth cells in the intestine. In addition, antigen-presenting cells (APCs) stimulate the production of IL-17in response to stress proteins, pathogen-associated molecular patterns (PAMPs), or microbial metabolites. IL-17 receptor (IL-17R) family is composed of IL-17RA, IL-17RB, IL-17RC, IL-17RE, as well as IL-17RA and C heterodimeric complex.

IL-17A can be secreted by other cell subsets, such as γδT cells, cytotoxic CD8^+^ T cells, innate tissue-specific cells, innate lymphoid cells (ILCs), and myeloid cells (Figure 2) (14, 15). IL-17A-mediated inflammation is required for host protection and survival against infection (8, 9, 16). IL-17A can also exacerbate fetal inflammatory responses, and has been implicated in immunopathology. IL-17A levels are elevated in various inflammatory conditions, including sepsis, pneumonia, systemic lupus erythematosus, rheumatoid arthritis, allograft rejection, and cancer (17, 18). Here we review the literature on IL-17-driven inflammatory and immune cascades during the development of sepsis.

## IL-17 Biology

### Structural Features of IL-17

The IL-17 family contains six isoforms of 20–30 kDa molecular weight and is a group of secreted and glycosylated proteins. All other members of the IL-17 family show 20–55% sequence homology to IL-17A, with IL-17E exhibiting the lowest homology with other family members (8, 9). IL-17A and IL-17F could exist as heterodimers or homodimers and are co-expressed by linked genes (19). Structurally, IL-17 family proteins have a conserved C-terminus with four cysteine residues, which form intramolecular disulfide bridges (20).

### Production of IL-17

IL-17 can be produced by a broad spectrum of cell populations (Figure 2), including Th17 cells, γδT cells, NKT cells, group 3 innate lymphoid cells (ILC3s), CD8^+^ (Tc17) cells, neutrophils, microglia, and mast cells (10, 12, 15). IL-23 and RORγt are indispensable for all IL-17-producing cell types (13).

Th17 cells were first identified in 2005 and were recognized as the primary source of IL-17 (16). In addition to IL-17, Th17 cells also produce a variety of inflammatory cytokines including granulocyte-macrophage colony-stimulating factor (GM-CSF), IL-21, and IL-22 (16). Th17-induced responses are implicated in host defense against infections, inflammatory and autoimmune disorders, and tumorigenesis (21). Other major sources of IL-17 include myeloid cells (e.g., kidneys and lungs) and Paneth cells in the intestinal crypts (8).

In response to stress proteins, pathogen-associated molecular patterns (PAMPs), or microbial metabolites, antigen-presenting cells (APCs) produce IL-23 and IL-1β to accelerate IL-17 release (8). IL-17 levels are depended on the particular context, including the pathogen and site and severity of infection (10, 12).

### IL-17 Receptor and Its Signal Transduction

IL-17 cytokines bind to five cytokine receptors (IL-17RA to IL-17RE, Figure 2) on target cells to drive their biological actions (2, 9). IL-17R is expressed in a variety of cell populations, including keratinocytes, fibroblasts, mesothelial cells, epithelial cells, and leukocytes (9). All these receptors share a SEFIR domain in the intracellular domain and a fibronectin III-like region in the extracellular region (10). IL-17RA is a shared receptor for different IL-17 isoforms. IL-17 cytokines can trigger signals via an IL-17RA/IL-17RC receptor complex. IL-17RB and IL-17RE serve as the specific receptors for IL-17B and IL-17RA/IL-17RB heterodimeric complex, respectively (9). IL-17A and IL-17F act through the same IL-17RA/IL-17RC receptor complex.

Studies suggest that IL-17RD also drives IL-17-mediated signaling, but the ligand of IL-17RD remains unknown (21).

**Figure 3:**
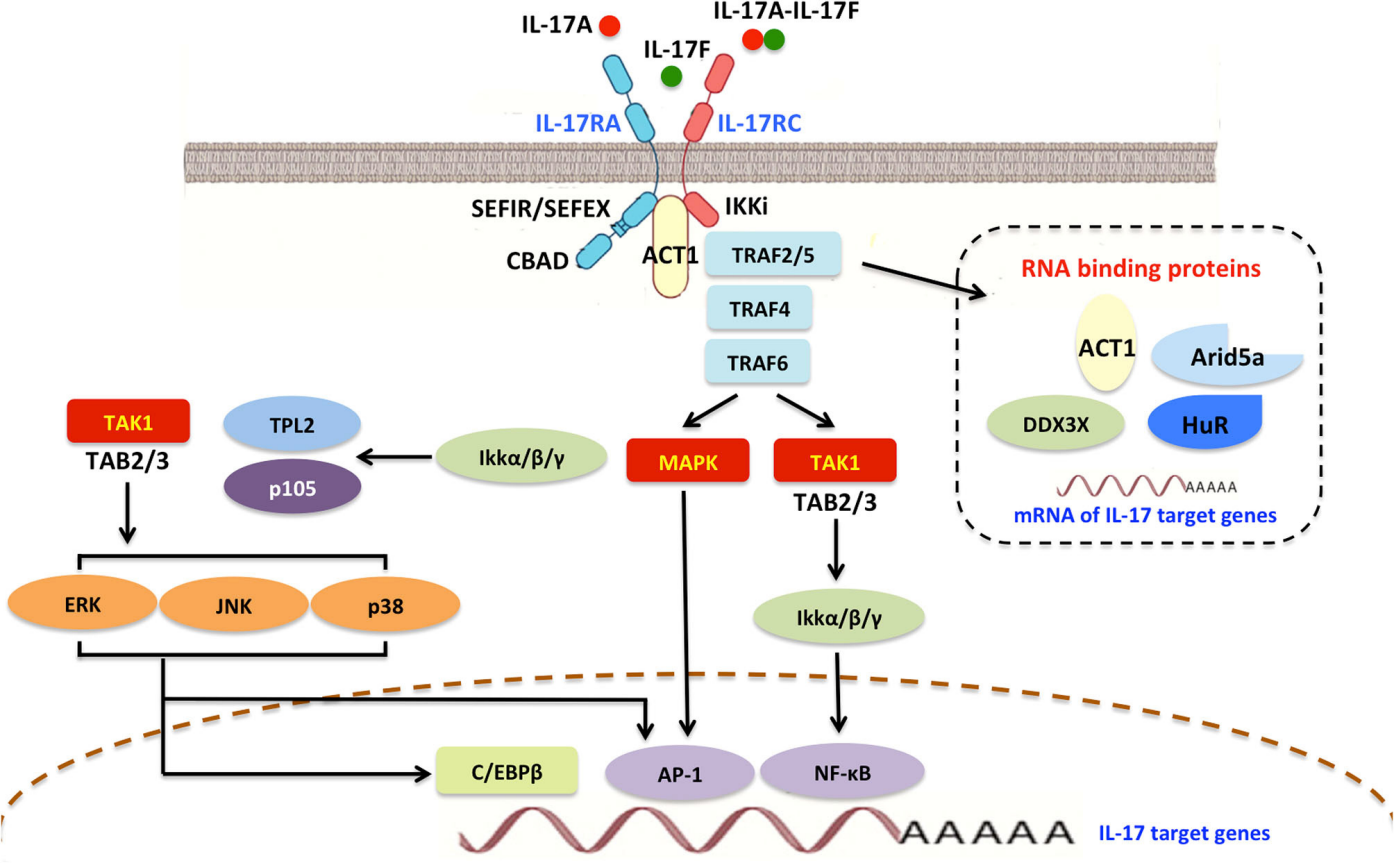
Interleukin-17A signaling transduction pathway. In general, all IL-17R members contain a shared and conserved cytoplasmic motif named a SEFIR/SEFEX domain similar to the TIR region in IL-1 receptors and TLRs. Initially, IL-17R signaling triggers the recruitment of Act1, which includes a SEFIR domain and is required for IL-17R-Act1 combination. Subsequently, Act1 can ubiquitinate TRAF6 and E3 ubiquitin ligase. Upon ligand binding, TRAF2/5, TRAF4, and TRAF6 can be engaged by Act1 and stabilize mRNAs by activating RNA-binding proteins such as Arid5a, HuR, and DDX3X. IL-17A promotes the activities of AP1 and MAPK, as well as δ transcription factors and C/EBPβ activation (CBAD). IL-17A also accelerates IκBα degradation and IKK activation, thereby inducing NF-κB signaling. NF-κB in turn improves IL-17A-mediated proinflammatory and anti-microbial responses. IL-17A/NF-κB signal transduction can trigger a feedback loop that controls overactivation of the NF-κB cascade. TRAF, TNF-receptor associated factor; SEFIR, similar expression of fibroblast growth factor and IL-17Rs; SEFEX, SEFIR extension; TLRs, Toll-like receptors; C/EBPβ, CCAAT/enhancer-binding protein β; CBAD, C/EBPβ activation domain; AP1, activator protein 1; MAPK, mitogen-activated protein kinase; NF-κB, nuclear factor κB; IKK, IκBα degradation and IκB kinase; JNK, Janus kinase; HuR, human antigen R.

IL-17A/IL-17C interacts with IL-17R to stimulate inflammatory reactions via activation of mitogen-activated protein kinase (MAPK), nuclear factor-kappa B (NF-κB), CCAAT/enhancer-binding protein (C/EBP), Janus kinase (JAK)/phosphatidylinostitol 3-kinase (PI3K), and JAK/STAT signaling (Figure 3) (22–25). IL-17A can also signal through Toll-IL-1 receptor (TIR)-domain-containing adapter-inducing interferon-β (TRIF), myeloid differentiation primary response gene 88 (MyD88), and the adaptor proteins (9, 21). Interestingly, IL-17A does not induce IL-17R cascades in embryonic fibroblasts of mice deficient in necrosis factor receptor-associated factor (TRAF)-6, suggesting that TRAF-6 is essential for IL-17A/IL-17R signaling (26). Furthermore, several studies have identified NF-κB activator 1 (Act1) as a key adapter molecule for TRAF-6 recruitment in the induction of IL-17R signaling (27).

### Functions of IL-17

#### IL-17A

IL-17A is the most studied member of the IL-17 family. IL-17A interacts with several mediators [e.g., GM-CSF, interferon (IFN)-γ, IL-22, IL-1β, tumor necrosis factor-α (TNF-α)] to exert its proinflammatory effect (15).

**Figure 4:**
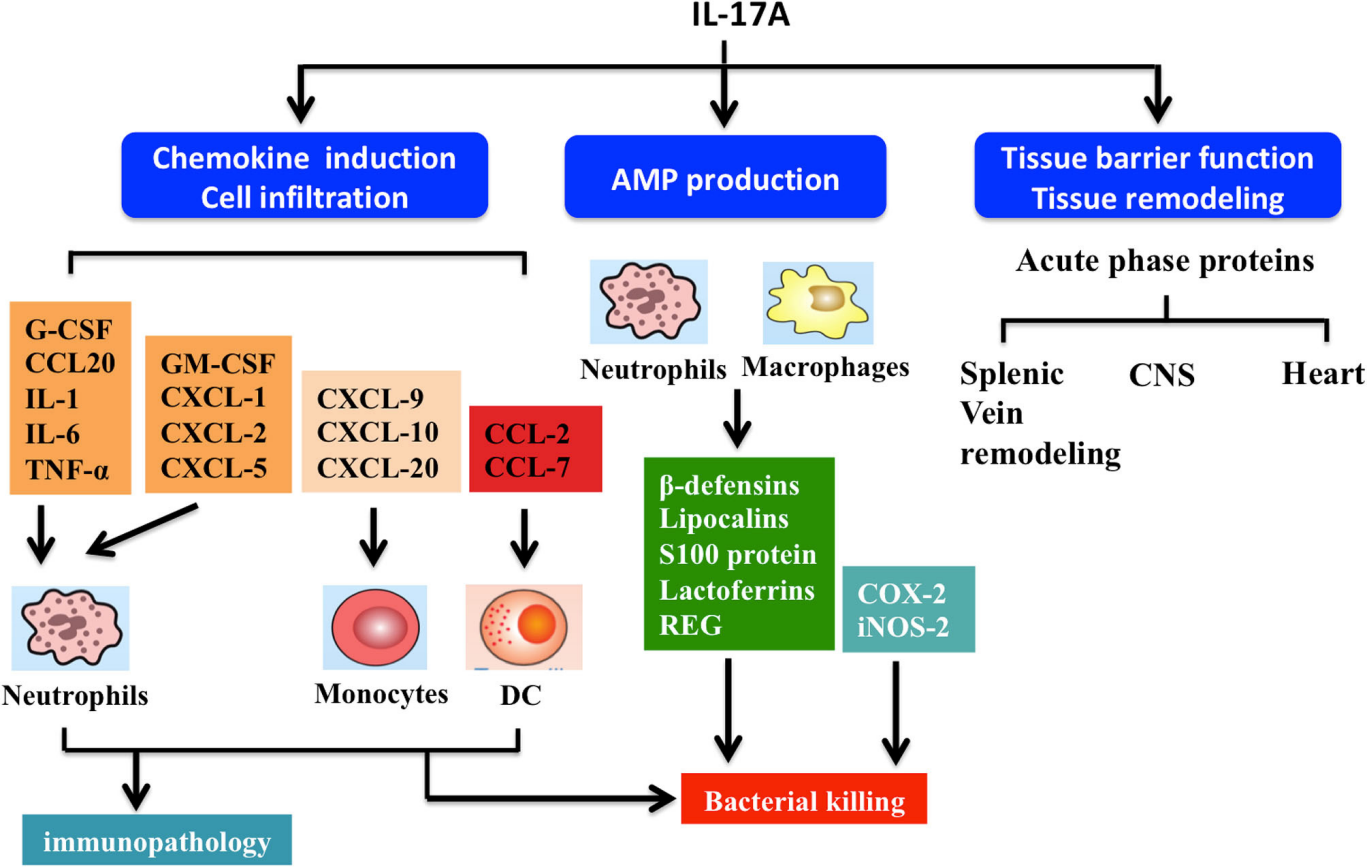
Summary of interleukin-17A functions. IL-17A-driven signaling drives several effector functions including chemokine induction, cell infiltration, antimicrobial peptide (AMP) production, and tissue barrier function and remodeling. Firstly, IL-17A triggers production of chemokine (C-C motif) ligand (CCL)-20 and granulocyte-colony stimulating factor (G-CSF), which act in synergy with other proinflammatory cytokines (e.g., TNF-α, IL-6, and IL-1) to induce neutrophil recruitment. IL-17A regulates the release of various chemokines. For example, chemokine (C-X-C motif) ligand (CXCL)-1, CXCL-2, CXCL-5, and granulocyte-macrophage colony-stimulating factor (GM-CSF) can improve neutrophil response. CCL7, CCL2, CXCL-9, CXCL-10, and CXCL-20 modulate the activities of monocytes and dendritic cells (DCs). Secondly, IL-17A stimulate macrophages and neutrophils to produce AMPs including β-defensins, lipocalins, S100 proteins, lactoferrins, and regenerating (REG) proteins, helping to kill bacteria. Thirdly, IL-17A signaling maintains tight junctions in mucosal tissues through formation of several acute phase proteins. IL-17A also participates in remodeling of the airway vascular, splenic vein, central nervous system (CNS), and heart tissues.

In general, IL-17A-mediated downstream pathways induce the production of inflammatory molecules, chemokines, antimicrobial peptides (AMPs), and remodeling proteins (Figure 4). IL-17A elicits crucial impacts on host defense, cell trafficking, immune modulation, and tissue repair, with a key role in the induction of innate immune defenses. IL-17A stimulates non-hematopoietic cells (e.g., epithelial cells) and then acts alone or synergistically with additional proinflammatory mediators to promote chemokine production [e.g., chemokine (C-C motif) ligand (CCL)-20, granulocyte-colony stimulating factor (G-CSF), C-X-C motif ligand (CXCL)-1, CXCL-2, and CXCL-8], thereby attracting myeloid cells to inflammatory sites (28). IL-17A also stimulates the release of IL-2 from Th cells, which in turn expands regulatory T cells (5). IL-17A also promotes the secretion of AMPs (e.g., β-defensins, calgranulin, S100A8, and lipocalin-2) from macrophages and neutrophils in response to acute pathogen invasion (21). Moreover, IL-17A may induce other AMPs and proteins, including inducible nitric oxide synthase (iNOS) and cyclooxygenase-2 (COX-2) (29).

IL-17A is critical for maintaining mucosal barrier integrity and function by increasing tight junctions and inducing acute phase proteins (8, 9). IL-17A helps to orchestrate airway vascular remodeling through Th17 cell responses in pulmonary inflammation (8). Similarly, IL-17A has been shown to upregulate endothelial tissue factor and is involved in splenic vein remodeling (30). Additionally, IL-17A plays a key role in repair and remodeling of other tissues such as ventricular tissue in myocardial infarction and bone resorption (31).

#### Roles of IL-17B Through IL-17F

IL-17B was originally identified as a proinflammatory mediator that accelerates neutrophil recruitment and migration (7). IL-17B inhibits IL-25 signaling and attenuates mucosal inflammation (5). IL-17B promotes the proliferation and survival of cancer cells in animal models (7), and increased IL-17B levels are linked to poor outcome in patients with several types of cancers (e.g., breast, lung, and pancreatic) (5).

IL-17C can be produced by several non-immune cells (e.g., epithelial cells, cutaneous neurons, and keratinocytes) and provide host protection in the intestine, skin, and the nervous system (5). For example, IL-17C released from epithelial cells maintains barrier integrity in an autocrine manner following epithelial damage (8). Emerging evidence indicates that IL-17C is sufficient to promote growth and survival of nerves and protect peripheral neurons during reactivation of herpes simplex virus-2 (6).

IL-17D is the least studied cytokine in the IL-17 family. Similar to other isoforms, IL-17D triggers secretion of diverse inflammatory cytokines such as GM-CSF, IL-6, and IL-8. Several studies have shown markedly increased IL-17D in viral infection and tumors (7).

Similar to IL-17A, IL-17E acts as a “mucosal barrier” molecule that confers immunity against parasitic infections (8). Thymic epithelial cells promote IL-17E production and host defenses via T cell receptor development of type 2 innate-like lymphoid cells (iNKT-2) (32). IL-17E is distinct from other member of the IL-17 family. IL-17E drives stromal cells, type 2 T helper (Th2) cells, epithelial cells, and ILC2s (5). It also accelerates production of thymic stromal lymphopoietin (TSLP), IL-13, IL-5, IL-4, and IL-13, and lowers the levels of IL-23, IL-6, and IL-1 (9). IL-17E is involved in the pathogenesis of parasitic and fungal infections, allergy, and autoimmune disorders. For example, large amounts of IL-17E are produced following infection with the parasitic helminth *Nippostrongylus* or *Aspergillus*. Furthermore, IL-17E-mediated responses depend on the airway epithelium, mast cells, eosinophils, and Th2 cells, thereby contributing to the immunopathogenesis of asthma (33).

IL-17F and IL-17A share similarities in receptors, signaling, function, and cellular sources. Similar to IL-17A, IL-17F plays a critical role in inflammatory responses and mucosal barrier maintenance (4).

## Roles of IL-17 in the Development of Sepsis

### IL-17 in Host Defense During Sepsis

**Figure 5:**
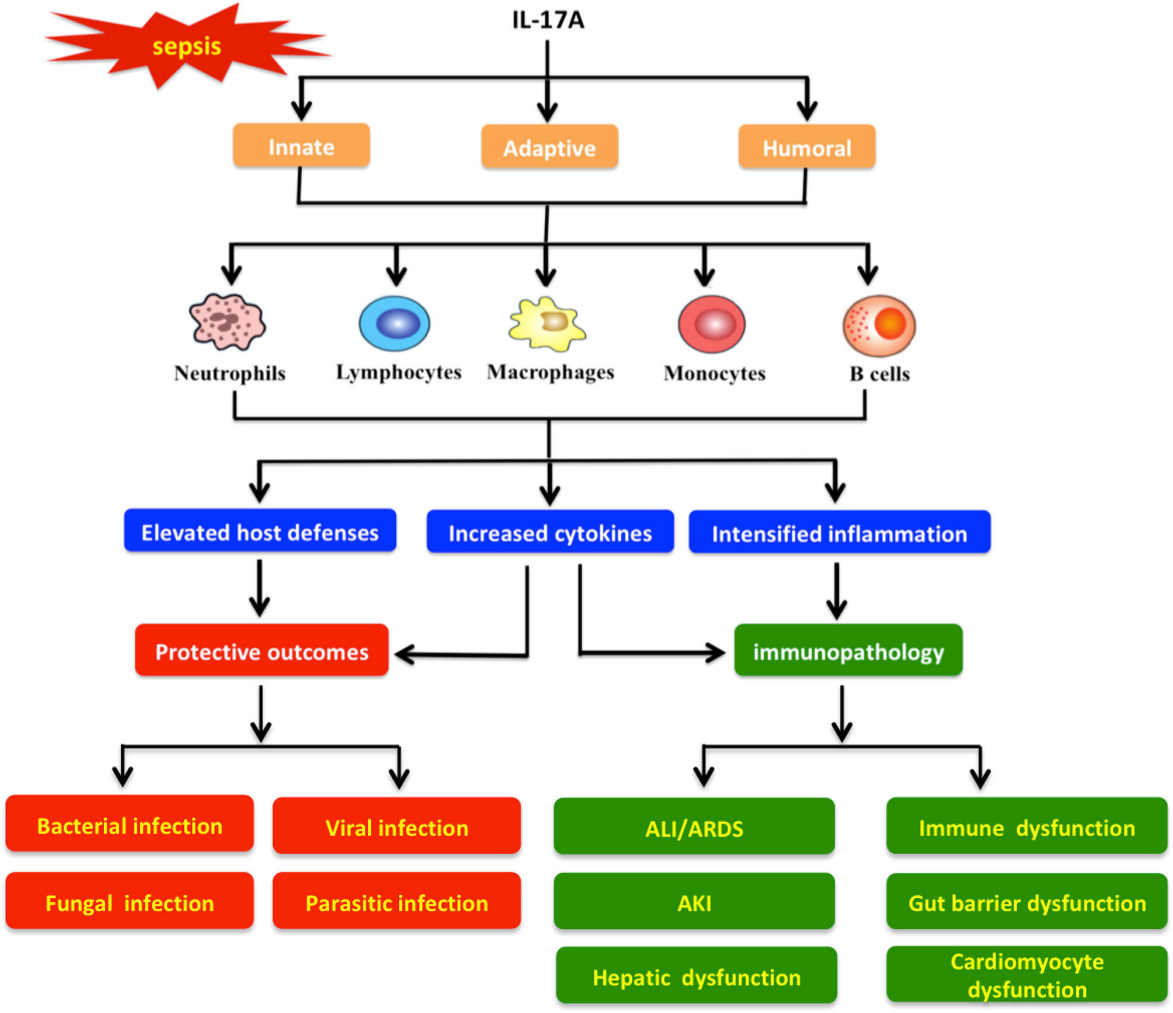
Protective and pathological roles of interleukin-17A in the pathogenesis of sepsis. IL-17A triggers various immune cells (e.g., neutrophils, lymphocytes, macrophages, monocytes, and B cells) to boost innate, adaptive, and humoral responses with different actions. Meanwhile, IL-17A signaling exhibits a strong protective effect against diverse infections (e.g., bacterial, fungal, viral, and parasitic infections) through promotion of host defenses and cytokine release. In addition, IL-17A may induce pathologic immune responses and then impair organ functions, which result in pathological alterations such as acute lung injury (ALI)/acute respiratory distress syndrome (ARDS), acute kidney injury (AKI), hepatic dysfunction, immune dysfunction, gut barrier dysfunction, and cardiomyocyte dysfunction.

Sepsis is caused by improper inflammatory and immune responses due to the inability of the host defenses to contain infection (34). As a crucial part of host immunity, IL-17A confers powerful protective effects against infections caused by bacteria, fungi, virus, and parasites (Figure 5); (Table 1) (16, 17). Increased circulating levels of IL-17A are observed in experimental and human sepsis (16, 17). A series of clinical studies have demonstrated that high serum IL-17A levels are associated with greater risk of sepsis, suggesting that this cytokine might serve as a novel predictor of sepsis progression as well as an attractive therapeutic target (34).

**Table 1:** Summary of studies on the pathophysiological significances of IL-17 in sepsis.

**System**	**Year**	**Authors**	**Clinical observations or conclusions**	**References**
Mice	2003	Tian et al.	CD4^+^ T cells mediated abscess formation in intra-abdominal sepsis by an IL-17-dependent mechanism	(35)
	2007	Shibata et al.	Resident Vδ1^+^ γδ T cells controlled early infiltration of neutrophils after *Escherichia coli* infection via IL-17 production	(36)
	2008	Flierl et al.	γδ T cell-derived IL-17 promoted high levels of proinflammatory mediators and bacteremia, increasing lethality	(37)
	2009	Freitas et al.	IL-17 enhanced the microbicidal activity of migrating neutrophils in sepsis induced by cecal ligation and puncture	(38)
	2010	Kasten et al.	IL-17 production by γδ T cells accelerated neutrophil recruitment in a sepsis model	(39)
	2012	Joshi et al.	Immunization with *Staphylococcus aureus* iron regulated surface determinant B conferred protection via Th17/IL-17 pathway	(40)
	2012	Ogiku et al.	IL-17A played a pivotal role in polymicrobial sepsis according to studies using IL-17 knockout mice	(41)
	2013	Bosmann et al.	Plasma concentrations of IL-17, IL-17F, and the IL-17AF heterodimer were obviously increased in mice after cecal ligation and puncture	(42)
	2014	Shimura et al.	IL-17, but not IL-17F or IL-25, was important to lipopolysaccharide-induced endotoxin shock; Myeloid cells and eosinohils, but not Th17 cells, was a source of IL-17 during endotoxin shock.	(43)
	2014	Jing et al.	Recombinant IL-17 rescued impaired host defense in *cxcl1^−/−^* mice; CXCL1 was important for IL-17 production via Th17 differentiation	(44)
	2014	Cauvi et al.	Elevated expression of IL-23/IL-17 pathway-related mediators correlated with exacerbation of pulmonary inflammation following polymicrobial sepsis	(45)
	2015	Costa et al.	Murine IL-17^+^Vγ4 T lymphocytes accumulated in the lungs and played a protective role in severe sepsis	(46)
	2015	Meng et al.	Activation of TLR2 by disseminated Gram-positive bacteria induced sustained upregulation of IL-17A and IL-6	(47)
	2016	Zhao et al.	Mice that completely lacked IL-17 failed to accumulate and activate neutrophils. Lung inflammation was attenuated in IL-17-deficient mice	(48)
	2016	Cen et al.	MFG-E8 downregulated IL-17 expression in sepsis by modulating STAT3 activation	(49)
	2016	Wynn et al.	IL-18 administration in sepsis increased IL-17A production by murine intestinal γδ T cells as well as Ly6G^+^ myeloid cells, and blocking IL-17A reduced IL-18-potentiated mortality in both neonatal sepsis and endotoxemia	(50)
	2016	Luo et al.	IL-17A knockout in mice protected against sepsis-associated acute kidney injury	(51)
	2017	Lv et al.	IL-33 attenuated sepsis by inhibiting IL-17 receptor signaling through upregulation of SOCS3	(52)
	2015	Szabo et al.	Rapid and rigorous IL-17A production by a distinct subpopulation of effector memory T lymphocytes constituted a novel mechanism of toxic shock syndrome immunopathology	(53)
Rats	2018	Han et al.	The levels of IL-17A in plasma, lung, and liver gradually increased with time in a sepsis neonatal rat model	(54)
Patients	2011	Makada et al.	IL-17A genetic variation was associated with altered susceptibility to Gram-positive infection and mortality in severe sepsis	(55)
	2011	Palumbo et al.	No significant associations were found between IL-6 and IL-17F genotypes and the related cytokine serum levels in burn patients with sepsis	(56)
	2015	Wu et al.	Treatment of anti-IL-17 enhanced IL-10 production but decreased IL-12 secretion in stimulated peripheral blood mononuclear cells of healthy controls and patients with severe sepsis	(57)
	2015	Paraschos et al.	Patients with multiple injuries showed defective TNF-α, IL-10, IL-17, and IFN-γ responses to a broad panel of bacterial stimuli	(58)
	2016	Maravistsa et al.	IL-17 was the only cytokine produced in high quantities by peripheral blood mononuclear cells and CD4^+^ lymphocytes in patients with septic shock and acute kidney injury	(59)
	2017	Preisser et al.	Increased IL-17 was noted in patients with sepsis-induced acute respiratory distress syndrome; IL-17 antibody administration might relieve acute lung injury symptoms by affecting RORγt levels and modulating the PI3K pathway.	(60)
	2017	Ali et al.	Elevated serum IL-17 increased the susceptibility for septic complications in polytrauma patients, and might be a useful biomarker of such risk	(61)

#### Bacterial Infection

It is widely accepted that bacterial infections predominantly contribute to the development of sepsis (18). Interestingly, IL-17A genetic variations influence the risk of gram-positive infection and correlate with short-term mortality in patients with severe sepsis (55). Studies suggest that IL-17A-mediated signaling orchestrates inflammatory and immune cascades by inducing proinflammatory mediators, thereby participating in preventing bacteria during sepsis (21).

IL-17A levels are positively associated with tissue bacterial loads in mice infected with *Klebsiella pneumonia* (62). Physiological levels of IL-17A in type 3 innate lymphoid cells (ILCs) accelerate G-GSF production and neutrophil recruitment, protecting the host against sepsis (15). Experimental and human sepsis involves impaired neutrophil recruitment and migration, the extent of which is positively associated with disease severity (63). IL-17A augments the migration and microbicidal activity of neutrophils, as well as mobilization of T lymphocytes (64). IL-17A improves neutrophil recruitment and bacterial eradication via γδT cells in a murine model of sepsis (36).

Decreased levels of IL-17A are correlated with increased risk of bacteremia (62). In IL-17R-deficient mice infected with *Klebsiella pneumoniae*, levels of G-CSF, macrophage inflammatory protein-2 (MIP-2), and chemokines were reduced, contributing to decreased neutrophil infiltration and microbial clearance (62). In agreement with this observation, IL-17A-deficient mice are also more susceptible to infection by other bacteria, including *Bordetella pertussis, Citrobacter rodentium, Escherichia coli*, and *Staphylococcus aureus* (65, 66). IL-17R-deficient mice showed decreased neutrophil recruitment, augmented infection spread, and exacerbated inflammatory responses following cecal ligation and puncture (CLP) (48). IL-17A also stimulates epithelial cells to trigger antimicrobial responses against intracellular bacteria such as *Listeria monocytogenes, Mycobacterium bovis Bacillus-Calmette Guérin*, and *Salmonella typhimurium* (67–69).

#### Fungal Infection

Invasive fungal infection is increasingly frequent in septic patients and correlates with a high risk of mortality (70). Importantly, IL-17A exerts immunoprotective effects in antifungal defense via induction of AMPs, chemokines, and proinflammatory cytokines (71). Meanwhile, IL-17A is sufficient to activate NK cells through release of GM-CSF, thereby eliciting its fungicidal activity (72). IL-17R knockdown increases susceptibility to fungal pathogens during systemic candidiasis (70, 73). Notably, low IL-17A levels are related to impaired host immunity in clinical observation (74). IL-17A responses are required for controlling infection with fungal pathogens, including *Aspergillus fumigatus, Pneumocystis carinii, Cryptococcus neoformans*, and *Candida albicans* (73–78). On the other hand, IL-17C induces lethal inflammation by exacerbating secretion of proinflammatory cytokines, contributing to the development of systemic fungal infection (78). These paradoxical effects require further investigation.

#### Viral Infection

Sepsis often occurs in cases of immunosuppression, when it manifests as an increased susceptibility to opportunistic infections, especially by virus (18). Several studies have shown that IL-17 promotes host immunity against influenza infection (21). In mice challenged with influenza virus, lack of IL-17 was associated with increased mortality (79). Adoptive transfer of IL-17-producing cells protected mice against a lethal dose of influenza virus (80). Also, IL-17A participates in the immunopathogenesis of influenza A (H1N1)-induced acute lung injury (80). Likely, IL-17A-mediated response is correlated with disease severity following infections by Epstein-Barr virus (EBV), herpes simplex virus (HSV), respiratory syncytial virus (RSV), vaccinia virus, and hepatitis virus (81, 82). Depletion of IL-17R mitigates inflammation and decreases neutrophil influx, hindering influenza infection (82). IL-17A neutralization could reduce viral load and mortality during herpes simplex virus infection (82). Protective or pathological roles of IL-17 during viral infections remain controversial.

#### Parasitic Infection

Few studies have examined the potential role of IL-17A in host defense against parasites (5). Studies suggest that Th17 cells, the major source of IL-17A, could impair host defenses against *Echinococcus granulosus, Leishmania braziliensis, Toxoplasma gondii*, and *Trypanosoma cruzi* (83–87). In patients with toxoplasmosis, CD8^+^ and CD4^+^ cells could limit parasitic replication and invasion (86). High IL-17A levels are detectable in peripheral blood mononuclear cells from patients with *Leishmaniasis* (87). IL-17R deficiency leads to lower expression of CXCL1 and CXCL2 in the liver and spleen as well as less neutrophil recruitment in *Trypanosoma cruzi*-infected mice (83). In the case of *Leishmania* infection, IL-17A blockade reduces disease severity in a mouse model (88). IL-17A antibody neutralization reduces inflammation and improves survival in mice following *Toxoplasma gondii* challenge (86). Collectively, available evidence supports a pathological role for IL-17 in parasitic infections, but further work is needed to explore whether this depends on the host, parasite, or other factors.

In summary, IL-17 contributes to host protection against diverse infectious organisms during sepsis while inducing hyperinflammation with detrimental outcomes for the host under certain conditions. Further investigation on the role of IL-17 and the interplay with other immune factors needs to be conducted in clinical settings.

### IL-17 in Organ Dysfunction Resulted From Sepsis

Studies have shown that overproduction of IL-17 could exaggerate immune responses, which in turn may lead to impaired organ function (89) (Figure 5). In line with these observations, elevated levels of IL-17A have been detected in plasma and tissues during sepsis associated with multiple organ damage (37, 38, 54, 56–58, 61) (Table 1).

#### Acute Lung Injury or Acute Respiratory Distress Syndrome

Acute lung injury (ALI) and acute respiratory distress syndrome (ARDS) are early events in the course of sepsis (90). Neutrophil infiltration plays an integral part in ARDS. IL-17R signaling is proposed to regulate neutrophil trafficking into the lung to maintain host immunity (9). The addition of recombinant IL-17A into the airway causes the production of chemokines that recruit inflammatory and immune cells (48). In contrast, IL-17RA or IL-17A knockout decreases CXCL1 and G-CSF levels in the bronchoalveolar lavage fluid of mice (62). In line with this, hyperproduction of IL-17A is involved in uncontrolled pulmonary inflammation in mice infected with *Haemophilus influenza* (91). Interestingly, γδT lymphocytes (comprising the Vγ4 T lymphocytes) accumulate in the lungs of septic mice (46). Surprisingly, administration of anti-Vγ4 monoclonal antibody increased the mortality induced by CLP in septic mice (13, 46). In mice with lipopolysaccharide-induced ARDS, levels of IL-17A were elevated in plasma, lung tissue lysate, and bronchoalveolar lavage fluid (60). Consistently, patients with sepsis-induced ARDS show persisting high levels of IL-17A, suggesting that IL-17A is a biomarker to assess the severity and prognosis of diseases (60). Moreover, activation of the IL-23/IL-17 signaling produces harmful effects on sepsis-driven lung inflammation (45). Specifically, IL-17A expression, known to be induced by IL-23, is significantly increased in the lungs of septic mice induced by CLP (45). Neutralization of IL-17A ameliorates ALI and ARDS by modulating PI3K signaling and RORγt expression (60), suggesting that IL-17 could be explored as a therapeutic target for sepsis-associated ARDS.

#### Immune Dysfunction or Immunosuppression

Sepsis disrupts immune homeostasis (64). A study in septic patients showed that inhibition of IL-17A by specific antibodies could lead to immune depression by increasing IL-10 release and decreasing IL-12 production from peripheral blood mononuclear cells (57).

A previous study reported that CD4^+^ T cells mediated abscess formation in intra-abdominal sepsis in an IL-17A-dependent manner (35). Similarly, IL-33, a potent immunoregulator, has been shown to attenuate sepsis by suppressing IL-17A-mediated signaling via upregulation of suppressor of cytokine signaling (SOCS)-3 (52).

Toxic shock syndrome caused by *Streptococcal* and *Staphylococcal* superantigens mimicks sepsis in clinical presentation. It is characterized by overexpression of proinflammatory cytokines and pathological immune responses (53). Overproduction of IL-17A was observed in the early phase of toxic shock syndrome; in contrast, anti-IL-17A antibodies obviously reduced intestinal and hepatic impairment and mortality rate (53).

#### Acute Kidney Injury

Acute kidney injury (AKI) is the hallmark and a risk factor of sepsis; reversal of AKI correlates with lower risk of mortality (89). Several experimental and clinical studies support the importance of IL-17A secretion in AKI (51). Strikingly, IL-17A can function as a chemokine that recruits neutrophils to the kidneys (55).

IL-17A is associated with elevated levels of proinflammatory cytokines and accelerated tubular epithelial apoptosis in AKI (51). IL-17A is upregulated in animal models of acute tubular injury and cisplatin-induced AKI (59). In Th17 cells stimulated by heat-killed *C. albicans*, IL-17A levels significantly increased in patients with AKI after sepsis compared with healthy counterparts (92). In one study, Th17 cell activation was higher in patients who died from AKI following sepsis than in survivors, and the extent of activation correlated with AKI-associated inflammation (93). IL-17A knockdown can mitigate interstitial neutrophil infiltration and tubular impairment (51, 59). The intracellular receptor RORγt is required for IL-17A production, and RORγt deficiency protects against cisplatin-induced nephrotoxicity (92). IL-17A induces neutrophil migration through CXCL5, a chemokine known to be associated with higher risk of renal damage. Interestingly, high IL-17C expression has been noted in kidney epithelial cells following fungal infection (6), whereas lack of IL-17C in mice was associated with lower renal damage and improved survival (78). Altogether, the evidence supports that IL-17-induced responses potentiate AKI in septic patients.

#### Gut Barrier Dysfunction

Intestinal mucosa dysfunction remains a challenge in the management of sepsis (64). Sepsis-induced gut barrier dysfunction includes disrupted mucosal integrity, increased permeability of the epithelial lining, impairment of gut-blood barrier, and bacterial translocation (90).

IL-17A is important in maintaining the integrity of epithelial barriers (8). IL-17A-mediated pathological responses disrupt intestinal epithelial barrier function, increase gut permeability, and cause translocation of gut bacteria by inhibiting proliferation of enterocytes and inducing their apoptosis (8, 9). Disseminated gram-positive bacteria can upregulate IL-17A by activating Toll-like receptor (TLR)-2 on T cells and dendritic cells (47). Neutralizing IL-17 in septic mouse models protects gut barrier integrity, reduces systemic inflammation and bacterial dissemination, and lowers mortality (41).

#### Cardiomyocyte Dysfunction

Cardiomyocyte dysfunction is a complication that contributes to high mortality in sepsis patients (90). The molecular mechanisms underlying sepsis-induced cardiomyopathy are not well defined, but may involve a combination of endothelial disturbance, autonomic nervous system alterations, dysfunction of calcium regulation, oxidative and mitochondrial stress, extensive inflammation, and myocardial ischemia and reperfusion (I/R) injury (90). Up to now, no efficient treatment for sepsis-induced cardiomyopathy exists.

IL-17A levels are greatly increased in post-myocardial I/R injury, inflammation, and apoptotic responses (94). IL-17A accelerates the release of chemokines and proinflammatory mediators from fibroblasts, endothelial cells, leukocytes, and neutrophils (95). In viral myocarditis, neutralizing IL-17A with a monoclonal antibody attenuates myocardium inflammation, clinical symptoms, and disease progression (96). Knockdown of IL-17A in mice greatly mitigates cardiac disturbance as well as myocardial I/R injury and remodeling (97). Anti-IL-17A antibodies downregulate CCL3, CXCL1, and IL-6 in cardiomyocytes of mice with sepsis induced by CLP, suggesting that IL-17A contributes to sepsis-induced cardiomyopathy (96). Interestingly, high mobility group box-1 protein (HMGB1), a crucial late biomarker of lethal systemic inflammation in sepsis, stimulates IL-17A release during myocardial I/R injury (98). Conversely, HMGB1 inhibition remarkably lowers IL-17A levels and attenuates myocardial I/R injury (98). Therefore, the HMGB1/IL-17A axis may play a key role in myocardial dysfunction resulted from sepsis.

#### Other Sepsis-Related Complications

Hepatic dysfunction is a prominent feature of sepsis with crucial implications for survival, since it contributes to sepsis-induced multiple organ failure (89). IL-17 from γδ T cells protects the host from *Listeria monocytogenes* infection by augmenting bacterial clearance through the liver (67). Absence of IL-17A or γδ T cells in mice is associated with greater bacterial load and inflammatory lesions in the liver (67). However, another study showed that IL-22, but not IL-17A, could protect hepatocytes from acute liver inflammation (99). The precise role of IL-17 in sepsis-induced hepatic damage remains unclear and requires deeper investigation.

The stress response following sepsis involves production of glucocorticoids and catecholamines (90). Both glucocorticoids and catecholamines inhibit the expression and production of IL-17A in lipopolysaccharide-stimulated peritoneal macrophages (90). They also block c-Jun-N-terminal kinase, preventing IL-17A secretion (42).

Taken together, excessive IL-17A production disrupts immune homeostasis and contributes to the development and progression of sepsis. Nevertheless, the exact contribution of IL-17 to sepsis-induced dysfunction of the liver and other organs needs to be further explored.

### Administration of Exogenous IL-17 or Targeting IL-17 in Sepsis

IL-17A is an important link between the innate and adaptive immune processes (43, 49). IL-17A-deficient mice are more susceptible to sepsis than wild-type controls (37), and IL-17A blockade impairs peritoneal eradication of *E. coli*, indicating that IL-17A might prevent sepsis in certain settings (63). Consistent with this idea, IL-17A administration restores impaired immunity in CXCL1-knockout mice (44). In animals infected with *K. pneumoniae*, treatment with IL-17A promoted bacterial clearance and neutrophil recruitment by enhancing the production of G-CSF, IL-1β, TNF-α, and CXCL-2 (36). Likewise, IL-17A administration improved anti-bacterial immunity following challenge with group B *Streptococcus* and *Streptococcus pneumoniae* in mice (34). Neutralization of IL-17A promoted resistance to intra-abdominal abscess formation in mice challenged with *Bacteroides fragilis* or abscess-inducing zwitterionic polysaccharides (35). Neutralizing IL-17A with antibodies could also reduce the mortality of mice immunized with *S. aureus* iron regulated surface determinant B after bacterial challenge (40). Thus, IL-17A plays a pivotal role in prevention of infection progression and related infectious complications.

In agreement with the findings described above, inhibition of IL-17/IL-23 significantly improved survival (from 40 to 100%) in a mouse model of endotoxic shock (39). Likely, anti-IL-17A antibody reduces systemic levels of proinflammatory cytokines/chemokines and bacteremia, improving the survival rate (50). *E. coli* infection results in high levels of IL-17, and blockade of IL-17 attenuates neutrophil infiltration and improves bacterial clearance (63).

In a recent study, anti-IL-17 antibody mitigated the pathology and the mortality rate of mice infected with *Pseudomonas aeruginosa* (100). In a colitis mouse model, IL-17 neutralization attenuated immunopathology and bacteremia (5). In a neonatal sepsis mice model, treatment with IL-17A-neutralizing antibody mitigated IL-18-related disease deterioration (57). Targeting IL-17A enhanced IL-10 production in peripheral blood mononuclear cells following sepsis (57). This evidence supports the possibility of reducing sepsis mortality through antibody-mediated blockade of IL-17R or IL-17A (Table 2). Anti-IL-17A antibodies ixekizumab and secukinumab are currently being investigated in clinical trials in septic patients (8, 9).

**Table 2:** Summary of studies on potential applications of IL-17 targeting in sepsis.

**Years**	**Authors**	**Potential applications**	**References**
2003	Tian et al.	Administration of a neutralizing antibody specific for IL-17A prevented abscess formation during bacterial challenge in mice	(35)
2007	Shibata et al.	*In vivo* blockade of IL-17A significantly reduced neutrophil infiltration and impaired bacterial clearance in mice	(36)
2008	Flierl et al.	Neutralization of IL-17A *in vivo* reduced levels of systemic proinflammatory cytokines and chemokines, and bacteremia in mice	(37)
2012	Joshi et al.	Neutralizing IL-17A *in vivo* significantly increased mortality in iron regulated *surface determinant B* immunized mice	(40)
2014	Jin et al.	Recombinant IL-17A rescued impaired host defense in *cxcl1^−/−^* mice; CXCL1 was important for IL-17A production via Th17 differentiation	(44)
2015	Wu et al.	Treatment with anti-IL-17A enhanced IL-10 production but decreased IL-12 secretion from stimulated peripheral blood mononuclear cells of healthy controls and patients with severe sepsis	(57)
2015	Meng et al.	IL-17A neutralization protected barrier integrity and improves survival of septic mice	(47)
2016	Wynn et al.	Blocking IL-17A reduced IL-18-potentiated mortality to both neonatal sepsis and endotoxemia	(50)
2016	Luo et al.	IL-17A knockout in mice could protect against sepsis-associated acute kidney injury	(51)
2017	Lv et al.	IL-33 attenuated sepsis by inhibiting IL-17 receptor signaling through upregulation of SOCS3 in mice	(29)
2017	Szabo et al.	Rapid and rigorous IL-17A production by a distinct subpopulation of effector memory T lymphocytes constituted a novel mechanism of toxic shock syndrome immunopathology in mice	(53)
2017	Ding et al.	Increased IL-17A was observed in patients with sepsis-induced acute respiratory distress syndrome; IL-17 antibody administration could relieve acute lung injury symptoms by affecting RORγt level and the PI3K pathway	(60)

## Summary and Perspectives

IL-17A can regulate host defenses against invading pathogens by producing chemokines, AMPs, and proinflammatory cytokines. In this way, IL-17A forms part of the alarm signal by sentry-like immune cells to stimulate host defenses. On one hand, IL-17A interacts with various mediators (e.g., TNF-α, IL-1, IL-6) to induce tissue-infiltrating neutrophils to eliminate the invading pathogens. On the other hand, IL-17A may interact with other proinflammatory cytokines to drive exaggerated immune response, and contribute to the development of inflammatory and autoimmune diseases. There is burgeoning evidence that IL-17A participates in the pathophysiology of sepsis, with respect to regulation of inflammatory and immune responses. Consistently, elevated level of IL-17A is apparently related with disease severity in sepsis, suggesting a potential biomarker of prognosis in the clinical setting.

Moreover, developing IL-17A as a therapeutic target must consider the fact that IL-17A might be either protective or pathogenic, depending on the specific circumstances. Future work will be needed to deeply explore the pathophysiological mechanisms by IL-17A interfere with divergent immune responses during sepsis. In addition, studies are warranted to evaluate the other forms (IL-17B-IL-17F) in clinical settings.

## Author Contributions

YY conceptualized the study. YG and MH conducted the literature review and drafted the manuscript. All authors contributed to the article and approved the submitted version.

## Conflict of Interest

The authors declare that the research was conducted in the absence of any commercial or financial relationships that could be construed as a potential conflict of interest.

## Abbreviations

AMPs: antimicrobial peptides
APCs: antigen-presenting cells
C/EBP: CCAAT/enhancer-binding protein
CCL: chemokine (C-C motif) ligand
CXCL: C-X-C motif ligand
COX-2: cyclooxygenase-2
EBV: Epstein-Barr virus
GM-CSF: granulocyte-macrophage colony-stimulating factor
G-CSF: granulocyte-colony stimulating factor
HMGB1: high mobility group box-1 protein
IFN-γ: interferon-γ
IL: interleukin
iNOS: inducible nitric oxide synthase
iNKT-2: type 2 innate-like lymphoid cells
ILCs: innate lymphoid cells
I/R: ischemia and reperfusion
JAK: Janus kinase
MAPK: mitogen-activated protein kinase
NF-κB: nuclear factor-kappa B
NKT: natural killer T cell
PI3K: phosphatidylinostitol 3-kinase
PAMPs: pathogen-associated molecular patterns
RSV: respiratory syncytial virus
RORγt: RAR-related orphan receptor γ
SEFIR: SEF/IL-17R
STAT: signal transducers and activators of transcription
Th17: CD4^+^ T helper 17
TIR: Toll-IL-1 receptor
TRIF: TIR-domain-containing adapter-inducing interferon-β
TRAF: tumor necrosis factor receptor-associated factor
TNF-α: tumor necrosis factor-α
Th2: type 2 T helper
TSLP: thymic stromal lymphopoietin
MyD88: myeloid differentiation primary response gene 88.
